# Intestinal bacterial community composition of juvenile Chinese mitten crab *Eriocheir sinensis* under different feeding times in lab conditions

**DOI:** 10.1038/s41598-022-26785-9

**Published:** 2022-12-23

**Authors:** Yingkai Xu, Ziwei Huang, Baoli Zhang, Changyue Yu, Lisong Li, Xiaodong Li, Yingdong Li

**Affiliations:** Key Laboratory of Livestock Infectious Diseases in Northeast China, Ministry of Education, College of Animal Science and Veterinary Medicine, Shenyang Agricultural University, Dongling Road 120, Shenyang, 110866 China

**Keywords:** Microbiology, Environmental sciences, Gastroenterology

## Abstract

Feeding time is an important factor affecting the physiological activity and feeding rhythm of crustaceans. However, little is known about the factors and mechanisms contributing to variations in feeding time in aquatic species or their impacts. Moreover, the gut microbiome largely affects host physiology and is associated with diet. To investigate the effects of different feeding times on the composition of intestinal bacterial communities, high-throughput 16S rRNA sequencing was used to monitor the gut bacteria of the Chinese mitten crab *Eriocheir sinensis* over a 10-day period under different feeding times: 06:00 h, 12:00 h, 18:00 h, and 24:00 h. Weight gain of the day-fed groups was significantly higher than that of the night-fed groups. Two probiotics, *Akkermansia muciniphila* and *Faecalibacterium prausnitzii*, were detected in the intestines of crabs in the 12:00 group. In addition, the diversity and richness of the flora in the 12:00 group were slightly higher than those in the other treatment groups. These results collectively indicate that different feeding times change the intestinal flora composition of Chinese mitten crabs, and further identified specific feeding times associated with a more significant weight gain effect. Our findings provide important insights into improving farming strategies for Chinese mitten crabs.

## Introduction

Feeding strategies and feed varieties have an impact in aquatic organism on growth^1^ and metabolism^2^; however, research in this field has largely focused on the feeds themselves, while underestimating the importance of feeding strategies. Over long-term evolution, aquatic organisms have acquired a certain feeding rhythm in response to the periodic changes of environmental factors such as light^3^, temperature^4^, and feeding rhythm^5^. Appropriate feeding frequency and feeding time can significantly improve the growth rate of aquatic organisms^6^, reduce food waste^7^, and effectively improve immune function^8^. Feeding time is an important factor to consider in aquaculture, but is often overlooked and has thus received minimal research attention^9^. To date, studies have shown that the optimal feeding time of aquatic animals is closely related to their feeding rhythms. For example, the fish species *Acipenser dabryanus* exhibited a better immune response under night feeding than under day feeding^10^.

Gut microbes play an important role in the physiological processes of their host, including nutrient metabolism^11^ and inhibition of pathogenic microorganisms^12^. The homeostasis of the gut microbiota of aquatic species is closely related to the host’s diet^13^. While previous studies have assessed gut microbiota responses to dietary changes, less is known about how feeding behavior affects the gut microbiota.

Chinese mitten crab (*Eriocheir sinensis*) is the main freshwater crab species cultured along the Yangtze River of China owing to its delicious meat, high economic value, and good breeding efficiency^14^. With improvement of the breeding efficiency of Chinese mitten crab, the breeding scale has expanded in recent years, with the total output reaching 7.57 × 10^8^ kg in China in 2018^15^. However, there has been no study assessing the changes in the gut microbiome under different feeding times in this species. Therefore, the aim of this study was to detect such changes by high-throughput sequencing, followed by community diversity, species richness, and bioinformatic pathway analyses. These findings will improve our understanding of the links between feeding times and gut microbial composition in crustaceans. Furthermore, this study can have important implications for improving aquaculture production efficiency.

## Materials and methods

### Ethics statement

The animals used in this study were not an endangered or protected species. At present, a license is not required to capture *E. sinensis* in China’s rice paddies. All experiments were carried out in accordance with the guidelines for scientific purposes, animal care and use formulated by the Animal Ethics Committee of Shenyang Agricultural University. All efforts were made to reduce animal suffering as much as possible.

### Sample collection

In December 2021, 60 Chinese mitten crabs (6.71 ± 1.53 g) were collected from paddy fields in Panjin City, Liaoning Province, China. The crabs were transported to Shenyang Agricultural University Aquaculture Laboratory and evenly allocated to four tanks equipped with a circulatory system, with two partitions placed in each tank to divide it into three units (n = 5 crabs per unit). Crabs were reared in these tanks under conditions simulating the natural temperature (15 ± 5 °C) and light cycle (12 h light/12 h dark). The crabs were fed the same commercial formulated diet (Wellhope Agri-tech Co. Ltd., China) every 2 days during a 2-week temporary rearing period, with cessation of rearing within 48 h before the experiment. After the start of the experiment, crabs in each tank were fed 1.5% of the total crab weight once a day using an automatic feeder. Twelve tanks were assigned a separate feeding time: 06:00 h, 12:00 h, 18:00 h, and 24:00 h, respectively (three replicates for each feeding time). Feeding was discontinued on day 11 and sample collection was performed at 12:00 h. The intestines of three crabs in each tank were randomly collected, pooled as a single sample per tank, and three replicates were used for each treatment. The collected samples were immediately frozen in liquid nitrogen and sent to Shanghai Personal Biotechnology Co., Ltd. (Shanghai, China) for DNA extraction and Illumina sequencing.

### DNA extraction

Intestinal genomic DNA samples were extracted using the OMEGA Soil DNA Kit (D5625-01; Omega Bio-Tek, Norcross, GA, USA) following the manufacturer’s instructions. A NanoDrop ND-1000 spectrophotometer (Thermo Fisher Scientific, Waltham, MA, USA) and agarose gel electrophoresis were used to measure the quantity and quality of the extracted DNA, respectively.

### 16S rRNA gene amplicon sequencing

The V3–V4 region of the bacterial 16S rRNA gene was amplified by polymerase chain reaction (PCR) with the forward primer 799F (5′-ACTCCTACGGGAGGCAGCA-3′) and reverse primer 1193R (5′-TCGGACTACHVGGGTWTCTAAT-3′). The PCR mixture contained 1 μL of DNA template. PCR was run on an Applied Biosystems 2720 thermal cycler (Invert Logan, Carlsbad, CA, USA) under the following thermal cycling conditions: initial denaturation at 98 °C for 5 min; followed by 25 denaturation cycles at 98 °C for 30 s, annealing at 53 °C for 30 s, and extension at 72 °C for 45 s; and a final extension at 72 °C for 5 min.

### Bioinformatics and statistical analysis

Microbiome bioinformatics was performed using QIIME2 2019.4 as described in the official tutorial (https://docs.qiime2.org/2019.4/tutorials/), with minor modifications. Raw sequence data were demultiplexed using the demux plugin, followed by primer trimming using the cutadapt plugin^16^. Reads were then filtered, denoised, and merged, and chimeric sequences were removed using the DADA2 plugin^17^. We used QIIME2 and the R package (v3.2.0) for sequence data analysis. Alpha diversity indices (Chao1, observed species, Shannon diversity index, Simpson index) at the amplicon sequencing variant (ASV) level were calculated using the ASV table in QIIME2 and visualized using boxplots. Abundance curves were generated at the ASV level to compare the abundance and homogeneity of ASVs in a sample. Beta diversity was analyzed to investigate structural changes in microbial communities between samples and visualized according to the principal coordinate analysis (PCoA) plot, multidimensional nonmetric scaling (NMDS), and hierarchical grouping by the arithmetic mean^18^. Using the R package "Venn Diagram," a Venn diagram was generated to show the shared and unique ASVs in a sample or population, based on the occurrence of ASVs in different samples and populations, regardless of their relative abundance^19^.

Network analysis based on relationships among microbial members was further applied as a common method of microbial community analysis^20^. The underlying purpose of this analysis is to find, through correlation analysis, the intrinsic patterns of co-occurrence or co-exclusion in specific microbial communities driven by spatiotemporal changes and environmental processes. Co-occurrence network analysis was performed using SparCC analysis^21^ with pseudo-count values between 10 and 6. The cut-off value of the correlation coefficient based on random matrix theory was determined to be 70 using the method implemented in the R package RMThreshold. The network was visualized using the R packages igraph and ggraph. A network hub is considered when the connectivity value within a module (Zi score) is greater than 2.5 and the connectivity value between modules (Pi score) is greater than 0.6^22^. Microbial function was predicted using PICRUSt2 (a phylogenetic survey of communities by reconstructing unobserved states)^23^ along with annotation in the MetaCyc and KEGG databases^24,25^.

### Ethics

The protocols for samples collection of crabs were approved by the Ethics Committee of Shenyang Agriculture University.

## Results

### Effects of different feeding times on growth performance of Chinese mitten crab

With the change of feeding time, the weight gain rate of Chinese mitten crab gradually decreased (Table 1), reaching the highest value of approximately 8.51% in the 06:00 h group, which was higher than that of the other three groups (P < 0.05). The weight gain rate of the 18:00 h group was the lowest, at approximately 3.36%.

**Table 1:** The impact of feeding time on the growth performance of Chinese velvet crabs.

Feeding time	06:00	12:00	18:00	24:00
Initial weight/g	6.58 ± 1.36	6.80 ± 1.79	6.84 ± 1.60	6.63 ± 1.30
Final weight/g	7.14 ± 1.48	7.25 ± 1.96	7.07 ± 1.61	6.99 ± 1.29
Weight gain rate/%	8.51 ± 0.08^a^	6.62 ± 0.09^b^	3.36 ± 0.06^c^	5.43 ± 0.007^d^

Weight gain rates with different superscripts are significantly different between feeding times (Duncan Test, p < 0.05).

### Sample bacterial sequencing

A total of 1,210,030 original sequences were read from the intestinal samples after sequencing, with an average of 100,835 sequence reads per sample. After quality filtering and denoising, 1,126,296 valid sequences remained (Table S1). We identified a total of 8804 ASVs at different taxonomic levels: 106 at the phylum level, 2816 at the genus level, and 843 at the species level (Table S2).

### Alpha diversity

The alpha diversity index was used to judge bacterial richness and diversity at the four feeding time points (6:00 h, 12:00 h, 18:00 h, and 24:00 h) (Fig. S1). There was no significant difference in the richness and diversity of the four groups of gut bacterial samples. Comparison of the Chao1, Faith_pd, and Observed_species values showed consistency in the indices with respect to the order of the four different time points, in which the flora diversity and richness in the 12:00 h group were slightly higher than those of the other three feeding time points. The sparse curve and abundance curve respectively illustrate the richness and diversity of each sample (Figs. S2 and S3), which both supported this finding.

### Abundance analysis

**Figure 1:**
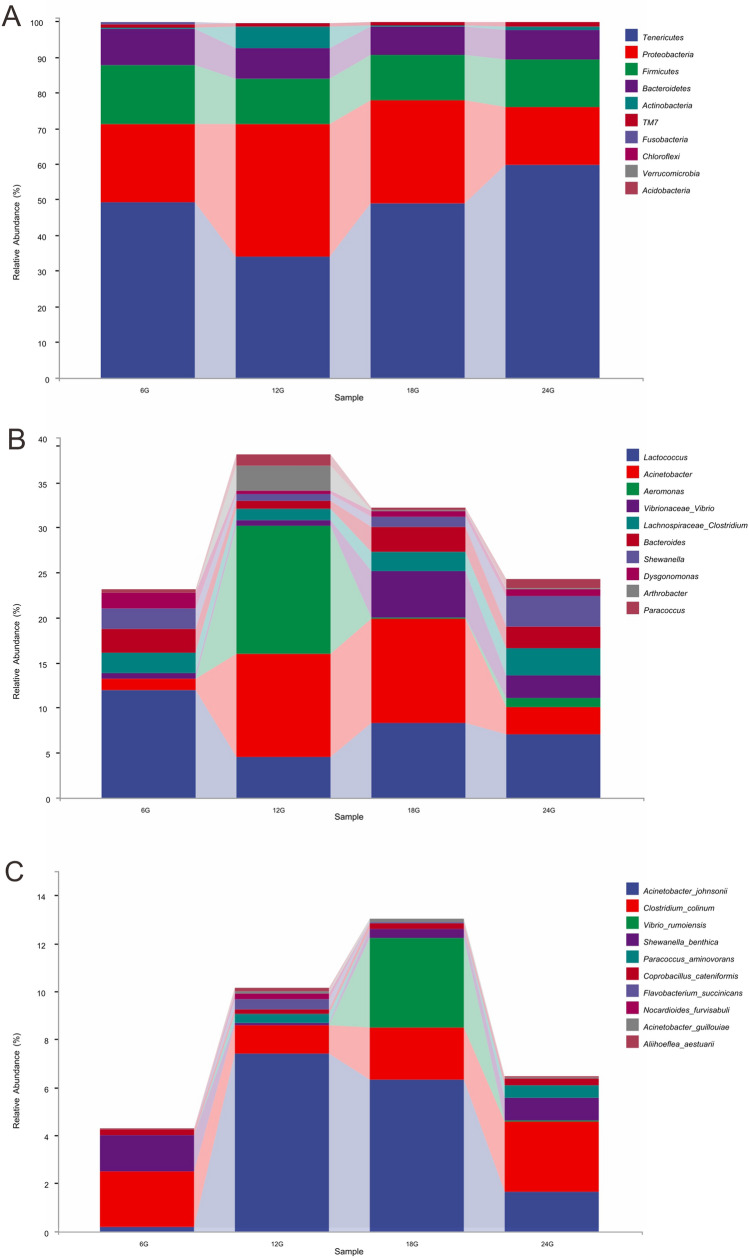
Relative abundance of gut microbial communities in *Eriocheir sinensis* at different feeding times at the phylum level (**A**), genera level (**B**), and species level (**C**).

At the phylum level, a total of four main phyla (> 1% relative abundance) were identified in the intestinal samples of *E. sinensis* subject to different feeding times, namely Proteobacteria, Firmicutes, Tenericutes, and Bacteroidetes. The abundance of these dominant phyla accounted for more than 90% of the entire flora (Fig. 1A). Among these dominant phyla, Tenericutes accounted for the highest proportion of bacteria in the intestinal tracts of crabs fed at 24:00 h (59.70%), but accounted for the lowest proportion in those fed at 12:00 h (33.95%). However, the proportions of Proteobacteria in each group showed completely opposite trends*,* accounting for the highest proportion in the intestines of crabs fed at 12:00 h (37.02%) and the lowest proportion in the intestines of crabs fed at 24:00 h (16.31%). The relative abundances of the two other dominant bacterial phyla were less affected by different feeding times, with relatively consistent proportions in each group.

At the genus level, *Acinetobacter* accounted for the highest proportion in the crab intestines fed during the day, at 11.39% and 11.60% in the 12:00 h and 18:00 h group, respectively. Moreover, we detected a significantly higher proportion of *Aeromonas* in the intestinal samples of crabs fed at 12:00 h compared with that detected in the other three groups (Fig. 1B).

The most abundant species were *Acinetobacter johnsonii* and *Clostridium colinum*. In addition, *Vibrio rumoiensis* was only detected in the guts of crabs fed at 18:00 h (Fig. 1C). These results indicated that feeding time has a certain influence on the composition of the intestinal flora of *E. sinensis*.

### Taxonomic differences and biomarkers

The number of gut bacteria was significantly different between groups with different feeding times (Fig. S4), ranked from the highest to the lowest as follows: 12:00 h (n = 1865), 4:00 h (n = 1201), 18:00 h (n = 899), and 06:00 h (n = 668). This result further supported that feeding time can alter gut bacterial communities.

**Figure 2:**
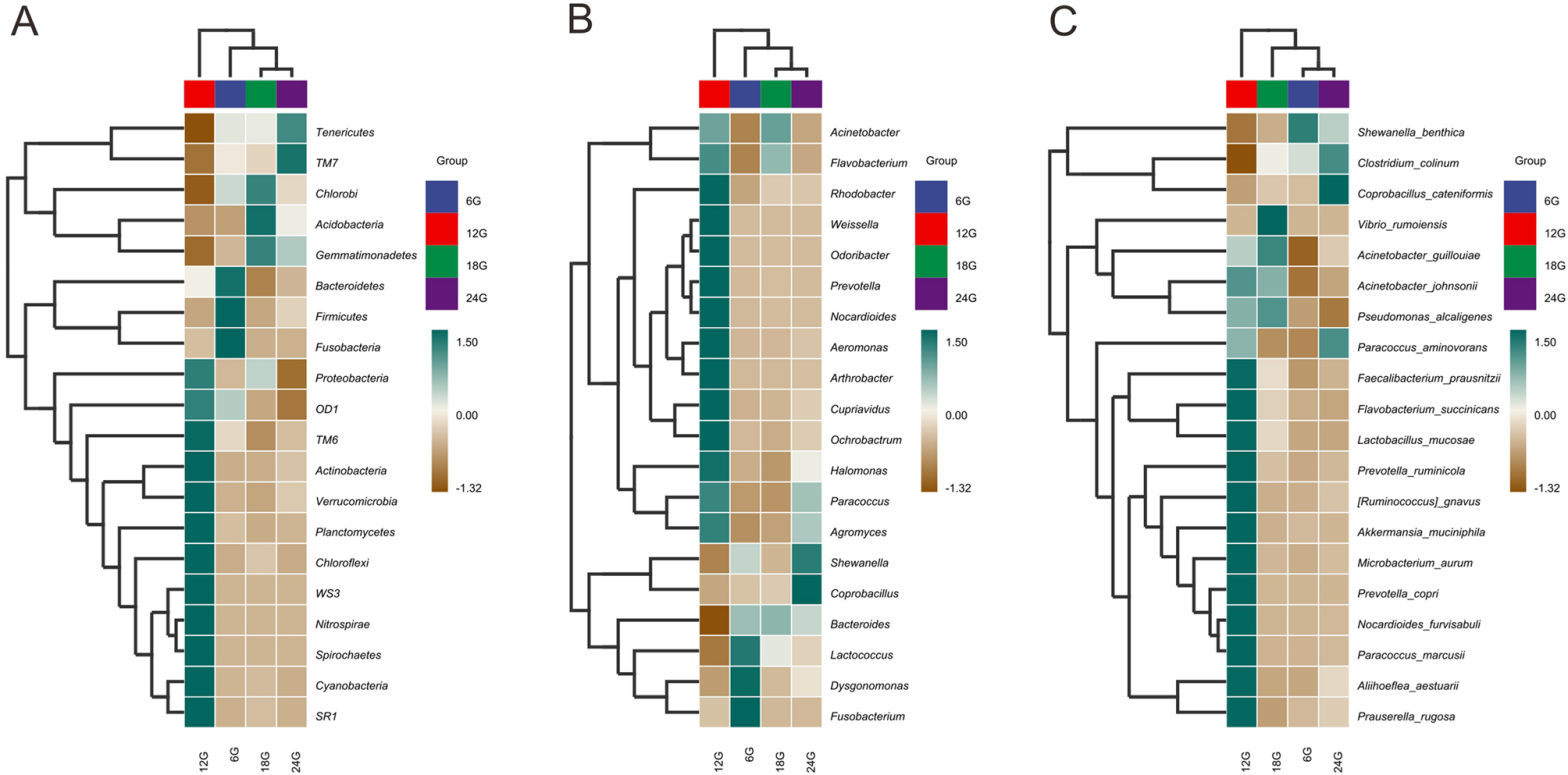
Correlation analyses of the gut bacteria at the phylum level (**A**), genera level (**B**), and species level (**C**).

The species composition heatmap showed that TM6, Actinobacteria, Verrucomicrobia, Planctomycetes, Chloroflexi, WS3, Nitrospirae, Spirochaetes, Cyanobacteria, and SR1 had high abundance only in the 18:00 h group samples (Fig. 2A). In addition, the relative abundances of Bacteroidetes, Firmicutes and Fusobacteria in the 06:00 h group were significantly higher than those of the other three groups. Similar abundance proportions were exhibited in the genus-level heatmap (Fig. 2B). Among the top 20 genera by abundance ratio, high abundance of *Dysgonomonas* and *Fusobacterium* was observed only in samples from the group fed at 06:00 h. Samples from the 12:00 h group had the highest genus abundance (13 out of 20 genera), including *Weissella* and *Odoribacter*. The heatmap exhibiting the abundance of the top 20 species showed that half of the species exhibited high abundance only in the 12:00 h group (Fig. 2C).

### Beta-diversity reveals changes in the gut microbiota under different feeding times

**Figure 3:**
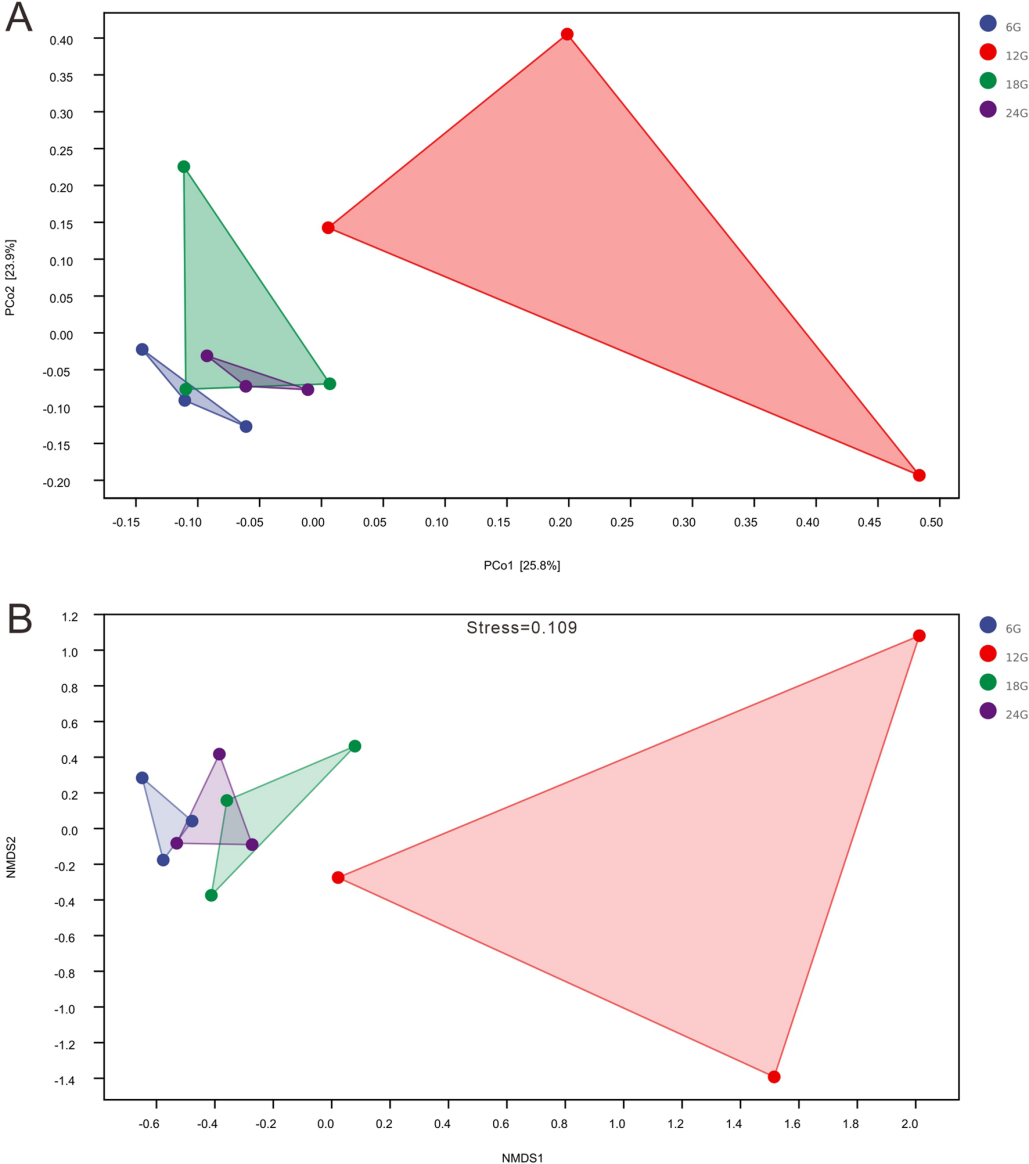
(**A**) Principal coordinate analysis (PCoA) of the gut bacterial community. (**B**) Nonmetric multidimensional scaling of the gut bacterial community.

Beta-diversity refers to differences in species composition or species replacement rates along an environmental gradient between different communities; therefore, it is also referred to as between-habitat diversity. We obtained the β-diversity index (Bray–Curtis distance) using PCoA and NMDS (Fig. 3A,B), which indicated that the microbial composition of the 12:00 h group was significantly different from that of the other groups. In contrast, within-group diversity was very low. These results indicated that crabs fed at 12:00 h exhibited significant variation in their gut bacterial communities.

### Keystone species based on network analysis

**Figure 4:**
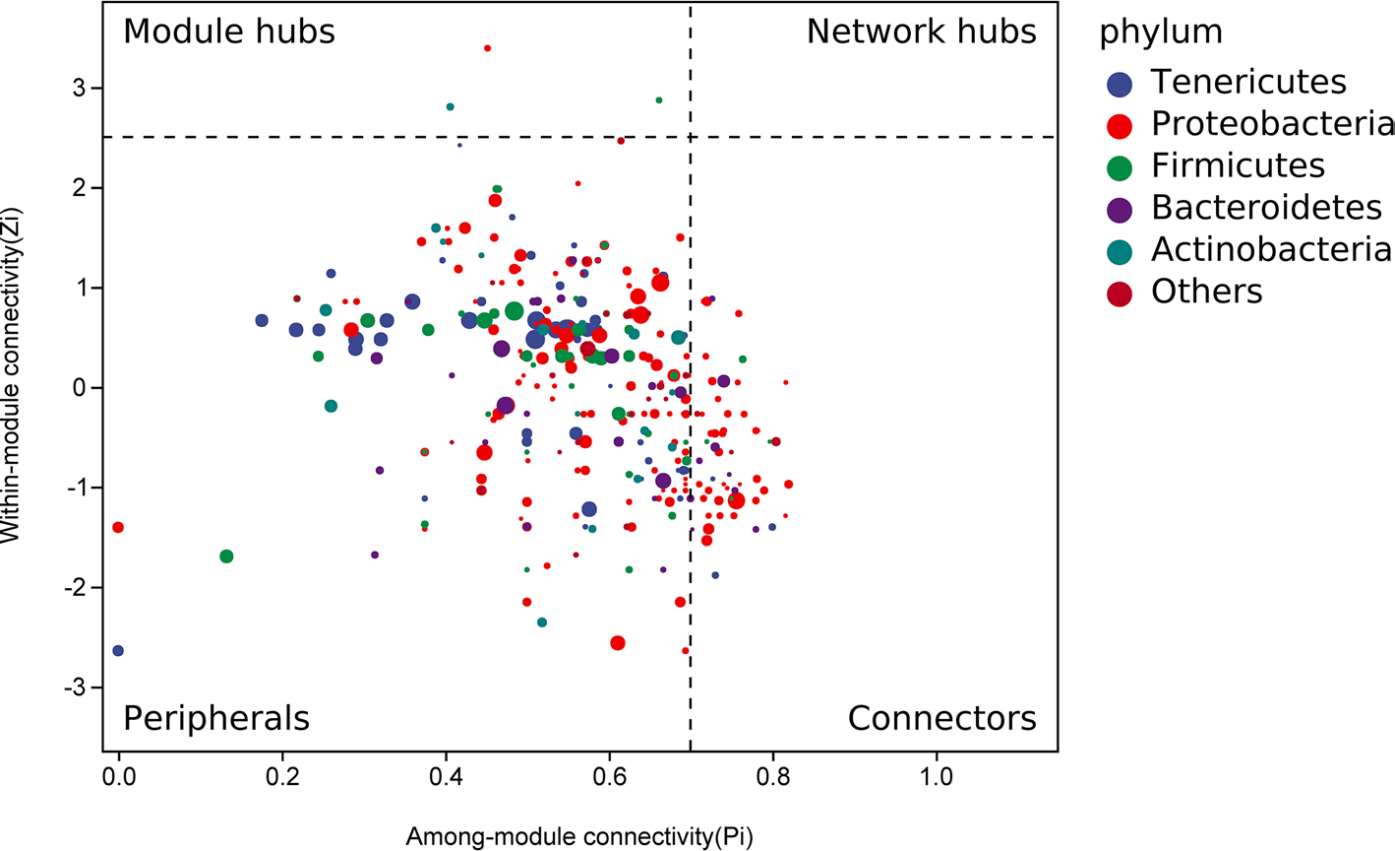
Network analysis of gut bacteria at the phylum level. The role of each node in the associated network is determined according to the values of Zi and Pi score. *Zi* module connectivity, *Pi* module connectivity.

Network analysis of the microbiota showed that Firmicutes (Zi = 2.8024, Pi = 0.4062) and Proteobacteria (Zi = 3.388, Pi = 0.4518) clustered close to generalists (Fig. 4). This suggests that changes in the ratios of these two phyla have the potential to affect the composition of the microbiome throughout the gut.

### Potential function of the gut bacterial community

**Figure 5:**
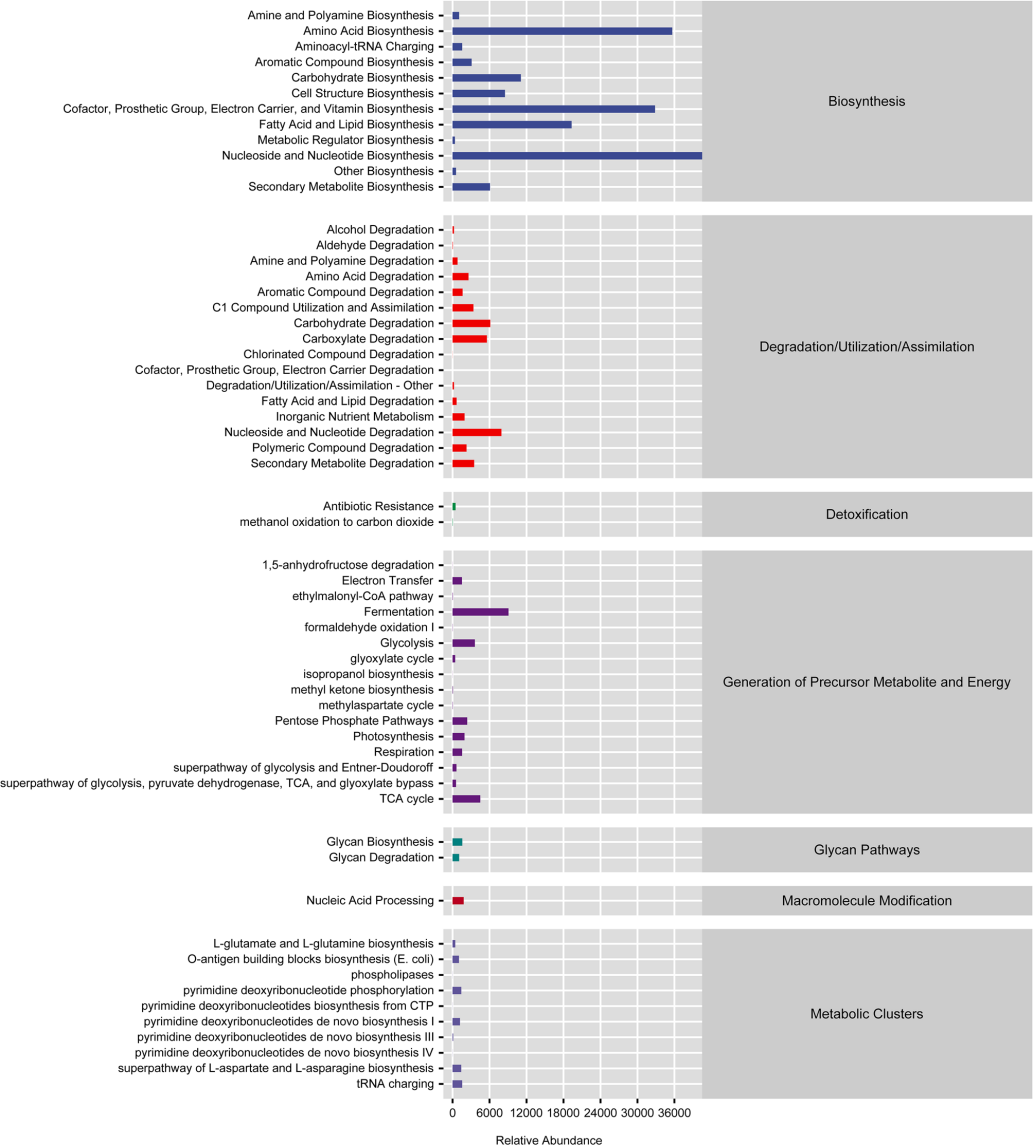
The relative abundance of metabolic pathways.

We determined the abundance of metabolic pathways active in the microbiome by consulting various metabolic pathway databases and applying calculation methods (Fig. 5). The biosynthesis pathway category had the most enriched pathways, including amino acid biosynthesis; cofactor, prosthetic group, electron carrier, and vitamin biosynthesis; fatty acid and lipid biosynthesis; and nucleoside and nucleotide biosynthesis.

## Discussion

Accumulating evidence indicates that diet affects the gut microbiome of aquatic animals to some extent^26^. Examining the modulation of gut microbiome composition and function is thus extremely helpful in understanding disease processes and identifying new therapeutic targets in aquatic animals^27^. To the best of our knowledge, this study is the first to investigate the changes of gut bacterial communities in *E. sinensis* under different feeding times. Our results showed that the composition of gut bacterial communities of *E. sinensis* at different feeding times markedly changed over a 10-day trial.

The results of the alpha- and beta-diversity analysis showed no significant difference in the composition of the intestinal bacterial community of each group of crabs after the 10-day experiment. Only the crabs fed at 12:00 h had a slightly higher diversity and richness of gut microbiota than those of the other groups. However, the species composition analysis showed distinct patterns of relative abundance of the dominant flora in each group.

Some of the bacteria with higher abundance in the group fed at 12:00 h were closely related to functions linked to the metabolism and immunity of aquatic animals. Two intestinal probiotics were detected among the 13 species with the highest abundance in the 12:00 h group, which are known to play crucial roles in the human gut^28,29^. *Faecalibacterium prausnitzii* has been reported as one of the major butyrate producers in the gut^30,31^, and Han et al.^32^ showed that supplementation of diets containing sodium butyrate can improve the intestinal integrity and immunity of juvenile Chinese mitten crabs. Feeding crabs a diet with a high concentration of sodium butyrate improved their survival rate and weight. In addition, related studies have demonstrated that *F. prausnitzii* and its metabolites play a protective role against colitis in mice^33^, improve an intestinal flora imbalance, and increase bacterial diversity and the abundance of short-chain fatty acid-producing bacteria. *Akkermansia muciniphila* mainly colonizes the outer mucus layer of the gastrointestinal tract, which uses the mucin in the gastrointestinal tract as an energy source for its own growth. Its consumption of mucin and goblet cell regeneration of mucin can achieve a dynamic balance, thereby maintaining stability of the host’s mucus layer^34^, which is essential for aquatic animals^35,36^. Damage to the intestinal mucus layer reduces the barrier effect against pathogens, which could facilitate infection of the host^37^. In addition, dietary supplementation of this probiotic has been associated with weight changes. *A. muciniphila* was found to be more abundant in the feces of lean mice compared with that in obese mice. Moreover, daily feeding of *A. muciniphila* for several weeks reversed high-fat diet-induced obesity in the mice, accompanied by improved epithelial integrity and related effects^38^.

When comparing the relative abundances of the 18:00 h group bacterial species, *Vibrio rumoiensis* was found to be the most abundant taxon. This result is consistent with our previous study involving a single-day microbiota analysis of Chinese mitten crabs^39^. However, many species in the present study were only observed to be highly abundant in the 12:00 h group, which may be due to changing the feeding time of the crabs. As a result, the feeding behavior of crabs was affected, which in turn resulted in a shifted bacterial flora composition. In addition, the weight gain rate of the 06:00 h and 12:00 h groups (i.e., the day-fed groups) was significantly higher than that of the crabs fed at night, whereas the number of ASVs in the 06:00 h group was lower than that of the two treatment groups fed at night. Nonetheless, these results are sufficient to indicate that feeding time has a certain effect on the composition of intestinal flora and the rate of weight gain of *E. sinensis*.

## Conclusions

Our evidence suggests that there is a certain degree of difference in the gut bacterial community composition of crabs with different feeding times, which provides a basis for further studies on the effects of feeding time in crustaceans. Therefore, in follow-up research, collecting samples from different breeding environments will be of great significance. In addition, the increasing trend of crab body weight in this study did not show a very complete cycle. Therefore, future research should consider adding more feeding modes to find the most suitable feeding time. Furthermore, our findings suggest that the timing of feeding may affect the gut microbial responses to oxidative stress and may even affect the innate immune response of *E. sinensis*. Taken together, our findings contribute to gaining a better understanding of the effects of different feeding times on the bacterial community structure in the gut of *E. sinensis*. These findings may in turn help to optimize feeding times to improve crab/crustacean quality and health in aquaculture environments.

## Supplementary Information

Supplementary Information 1.

Supplementary Information 2.

Supplementary Information 3.

Supplementary Information 4.

Supplementary Information 5.

Supplementary Information 6.

## Data Availability

The raw reads have been deposited into NCBI database (BioProject number PRJNA885001).
