# Supplementary figures and images for: Intestinal bacterial community composition of juvenile Chinese mitten crab Eriocheir sinensis under different feeding times in lab conditions
Source: Sci Rep. 2022 Dec 23;12:22206. doi: 10.1038/s41598-022-26785-9 (PMC9789113; doi:10.1038/s41598-022-26785-9)

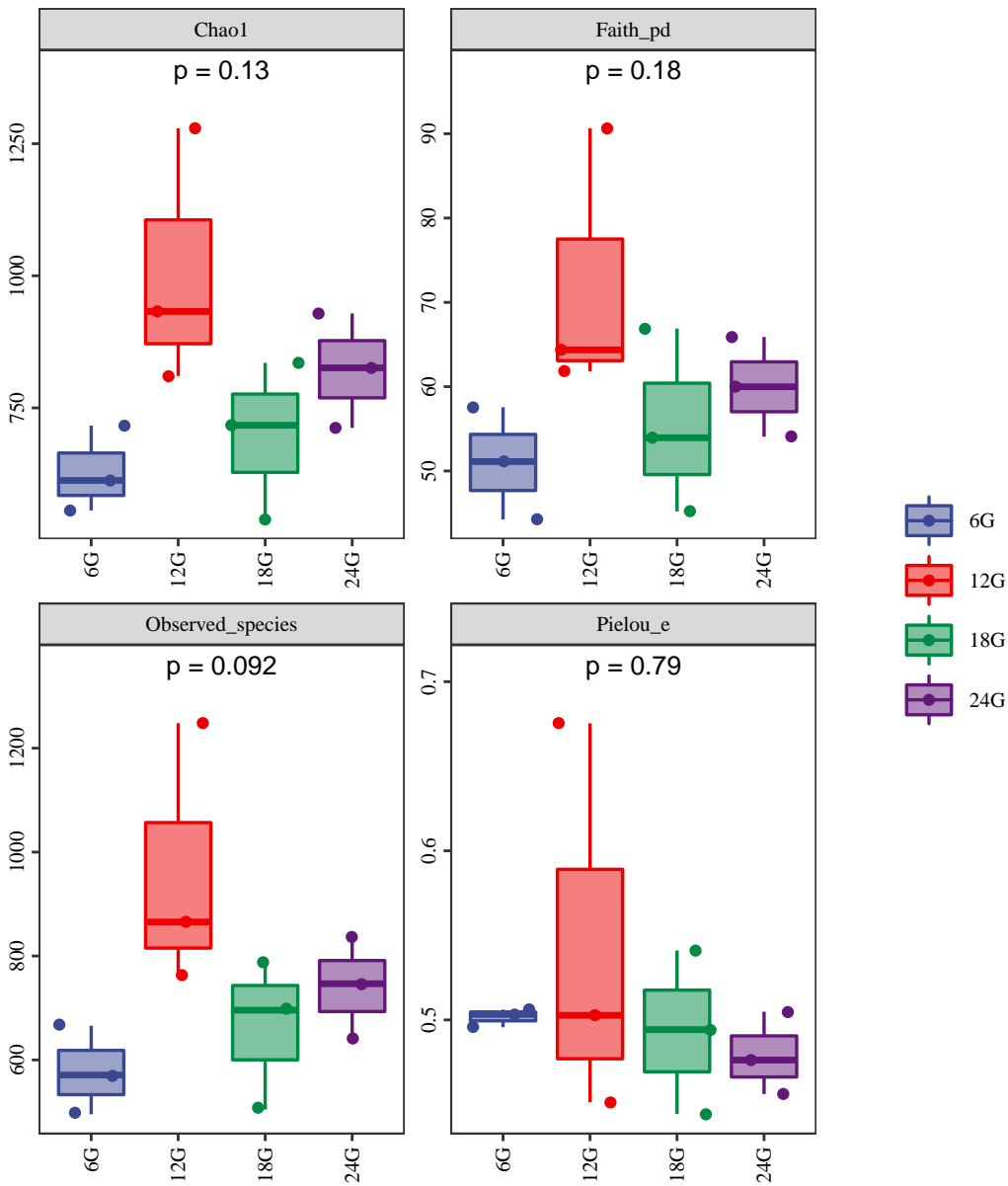

Supplement: Supplementary file 1 — Supplementary Information 1. [file 41598_2022_26785_MOESM1_ESM.pdf]

# Observed\_species

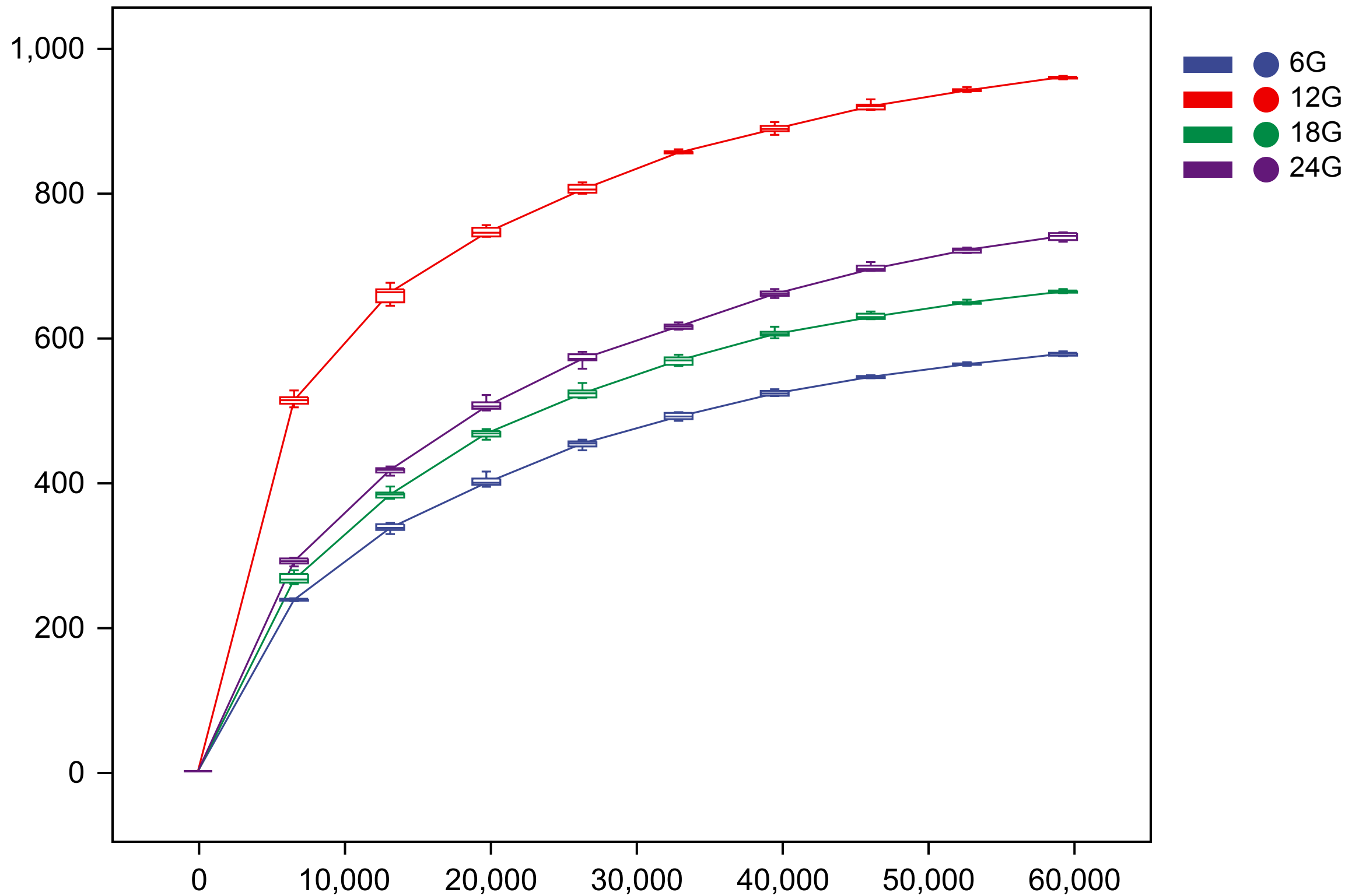

Supplement: Supplementary file 2 — Supplementary Information 2. [file 41598_2022_26785_MOESM2_ESM.pdf]

Rank Abundance Curve

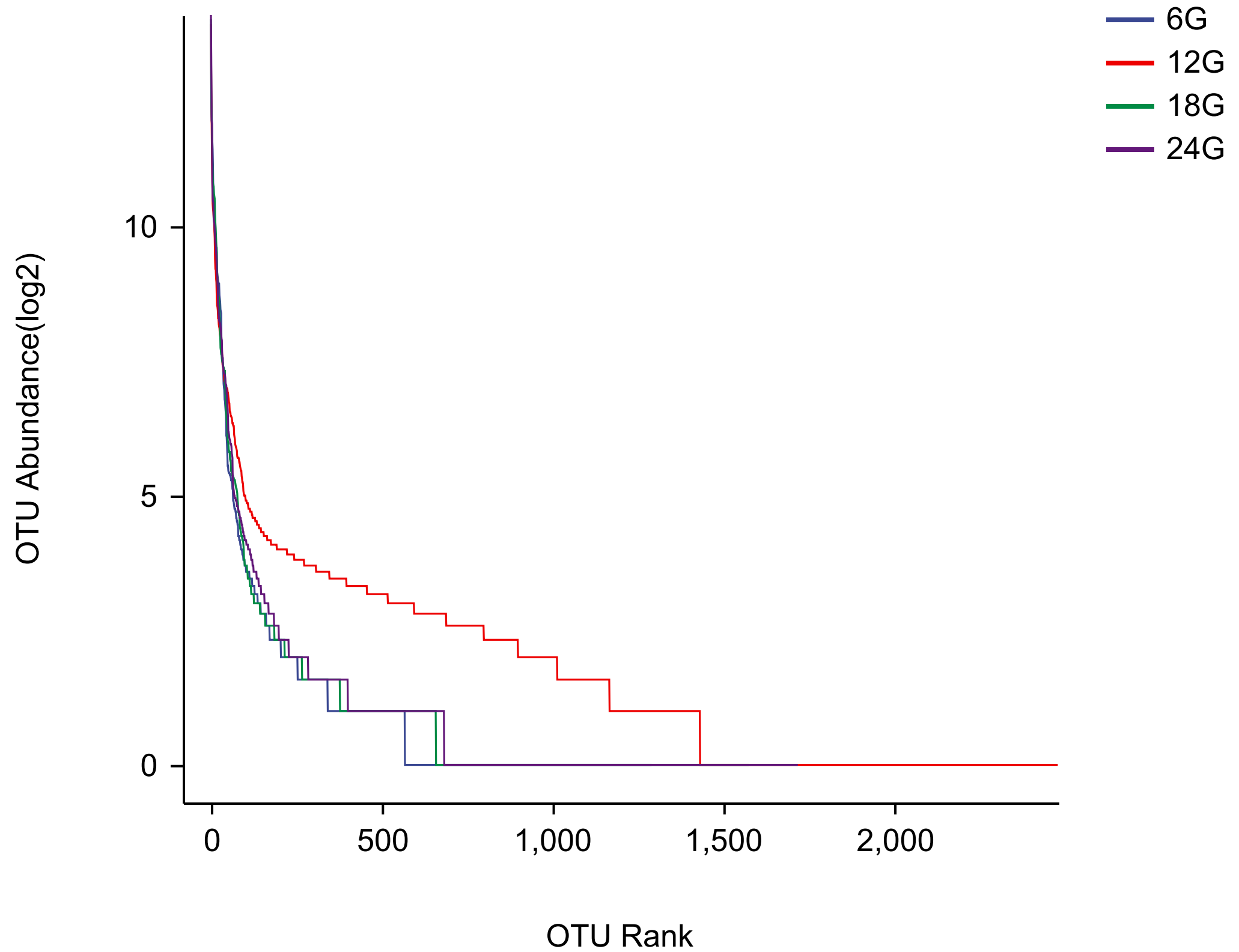

Supplement: Supplementary file 3 — Supplementary Information 3. [file 41598_2022_26785_MOESM3_ESM.pdf]

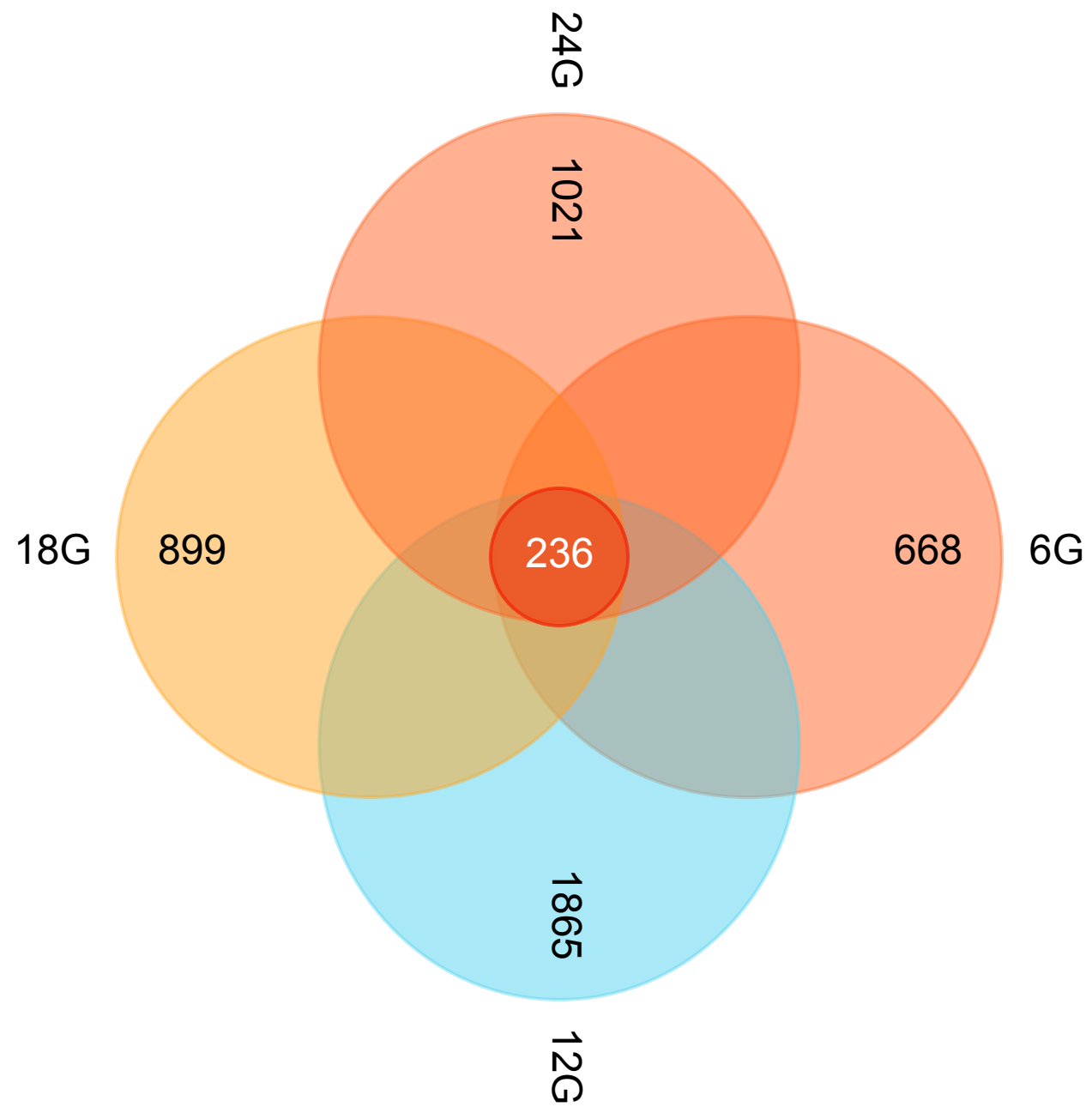

Supplement: Supplementary file 4 — Supplementary Information 4. [file 41598_2022_26785_MOESM4_ESM.pdf]
