# Supplementary material for: Intestinal bacterial community composition of juvenile Chinese mitten crab Eriocheir sinensis under different feeding times in lab conditions
Source: Sci Rep. 2022 Dec 23;12:22206. doi: 10.1038/s41598-022-26785-9 (PMC9789113; doi:10.1038/s41598-022-26785-9)
Supplement: Supplementary file 5 — Supplementary Information 5. [file 41598_2022_26785_MOESM5_ESM.docx]

**Supplement Figure legends**

Figure S1. Alpha-diversity indices (Chao1, Observed species, Faith_pd, Pielou_e) of the gut bacterial community. Box plots depict the medians (central horizontal lines), inter-quartile ranges (boxes), and 95% confidence intervals (whiskers). P-value are from Kruskal-Wallis test. Asterisks indicate statistically significant differences between pairs of values (*P < 0.05, **P < 0.01, ***P < 0.001, and ****P < 0.0001).

Figure S2. Sparse curves of intestinal bacteria in *Eriocheir sinensis* at different feeding times (Chao1, Observed species, Faith_pd).

Figure S3. Intestinal bacterial abundance grade curve of Chinese mitten crab at different feeding times.

Figure S4. Venn diagram of distribution of OTUs in intestinal bacteria of *Eriocheir sinensis* at different feeding times
