# Supplementary material for: Intestinal bacterial community composition of juvenile Chinese mitten crab Eriocheir sinensis under different feeding times in lab conditions
Source: Sci Rep. 2022 Dec 23;12:22206. doi: 10.1038/s41598-022-26785-9 (PMC9789113; doi:10.1038/s41598-022-26785-9)
Supplement: Supplementary file 6 — Supplementary Information 6. [file 41598_2022_26785_MOESM6_ESM.docx]

Table S1 Sequencing statistics of gut bacteria

| SampleID | Input | Filtered | Denoised | Merged |
| --- | --- | --- | --- | --- |
| 6_G_1 | 96563 | 92610 | 91490 | 86803 |
| 6_G_2 | 97739 | 93544 | 92574 | 90247 |
| 6_G_3 | 97197 | 93693 | 92464 | 90122 |
| 12_G_1 | 104616 | 99132 | 98051 | 96393 |
| 12_G_2 | 103074 | 97895 | 96531 | 92079 |
| 12_G_3 | 108203 | 89985 | 89009 | 82686 |
| 18_G_1 | 96909 | 92865 | 91348 | 88488 |
| 18_G_2 | 90916 | 85948 | 85090 | 83403 |
| 18_G_3 | 90037 | 85930 | 84668 | 81026 |
| 24_G_1 | 104342 | 99536 | 98497 | 95527 |
| 24_G_2 | 113152 | 106928 | 105515 | 103168 |
| 24_G_3 | 107282 | 102293 | 101059 | 98831 |

Table S2 Number of ASVs of different taxonomic levels in gut samples.

| ID | domain | phylum | class | order | family | genus | species |
| --- | --- | --- | --- | --- | --- | --- | --- |
| 6_G_1 | 41 | 8 | 118 | 101 | 135 | 116 | 54 |
| 6_G_2 | 36 | 4 | 143 | 69 | 87 | 119 | 41 |
| 6_G_3 | 38 | 10 | 141 | 110 | 133 | 186 | 48 |
| 12_G_1 | 31 | 4 | 118 | 87 | 163 | 366 | 90 |
| 12_G_2 | 49 | 8 | 96 | 97 | 155 | 253 | 97 |
| 12_G_3 | 27 | 9 | 79 | 185 | 349 | 490 | 112 |
| 18_G_1 | 41 | 10 | 136 | 140 | 176 | 220 | 69 |
| 18_G_2 | 35 | 5 | 102 | 87 | 92 | 144 | 37 |
| 18_G_3 | 44 | 11 | 97 | 86 | 131 | 242 | 81 |
| 24_G_1 | 39 | 12 | 122 | 85 | 100 | 221 | 60 |
| 24_G_2 | 49 | 9 | 147 | 105 | 141 | 213 | 77 |
| 24_G_3 | 54 | 16 | 126 | 137 | 179 | 246 | 77 |
